# The characterization of unidirectional and woven water hyacinth fiber reinforced with epoxy resin composites

**DOI:** 10.1016/j.heliyon.2022.e10484

**Published:** 2022-08-31

**Authors:** S. Sulardjaka, N. Iskandar, Sri Nugroho, A. Alamsyah, M.Y. Prasetya

**Affiliations:** a Mechanical Engineering Department, Diponegoro University, Jl. Prof. Sudarto, SH, Semarang, Indonesia; b Advanced Materials Laboratory, CoRES Diponegoro University, Jl. Prof. Sudarto, SH, Semarang, Indonesia

**Keywords:** Composite, Epoxy-resin, Water hyacinth, Woven fiber, Unidirectional fiber

## Abstract

The high growth of Water Hyacinth/*Eichhornia crassipes* (WH) led to several problems such as ecosystem, irrigation, and sedimentation. The rapid growth of water hyacinth in natural rivers, reservoir, lake and canals causes drainage problems in many nations. As a result, local offices must spend significant annual budgets to dispose of water hyacinth wastes. Meanwhile, cellulose fiber from WH had a potential application in natural fiber composite (NFC). This study investigated the development and use of water hyacinth wastes for the production of unidirectional dan weaved fiber epoxy resin composites. The purpose of this research is to investigate at the mechanical and physical properties of unidirectional WH and woven fiber reinforced epoxy resin composites in variation of 0 % wt., 15 % wt., 25 % wt. and 35 % wt. of WH fibers. The WH fiber was obtained from a mechanically processed WH plants. The composites were manufactured through the hand lay-up method. The tensile and impact tests were carried out based on ASTM D3039 and ASTM D6110 respectively, while the density of composites was tested based on the Archimedes rule. The results of this study showed that increasing of % wt. of the WH woven fiber, the tensile strength of composite decrease. The impact strength of composites increases by the rise of % wt. of the WH woven fibers. The % wt. of WH woven fibers was in direct proportion to the amount of pore or void between the fibers and matrix, which led to a delamination mode fracture. Tensile and impact strength of unidirectional WH fiber increase by increasing the % wt. of WH fibers.

## 1 Introduction

Natural fibers are a useful class of materials that are environmentally clean, renewable, and biodegradable resources. They are also used in manufacturing natural fiber composites with advantages which include low impact on the environment, renewability, inexpensive and easily degraded [1, 2, 3, 4]. However, the use of natural fibers as a reinforcement of composites is still experiencing several problems such as, low mechanical properties, hydrophilic properties, limited processing temperatures, low matrix and fiber binding forces that are easily degraded [5, 6]. Furthermore, studies on the development of natural fiber properties were carried out by pretreating or engineering the manufacturing method [7, 8].

Water hyacinth (*Eichhornia crassipes*) is a type of aquatic plant that floats on the surface of the water. It grows aggressively and was a nuisance on almost all continents for more than 100 years. Furthermore, its high population growth led to several problems related to ecosystem balance, decreased fish production, loss of endemic organisms and sedimentation [9, 10, 11]. Numerous studies were carried out to utilize water hyacinth plants as absorbers of heavy metals, absorbing dye waste, biofuel and biogas production, composite catalyst and reinforcing composites [12, 13, 14, 15, 16, 17, 18, 19].

Based on previous studies, the problem associated with the utilization of WH materials for composite reinforcement includes, low mechanical strength, ease of water absorption leading to a reduced fiber bond with the matrix and weak compatibility of the WH fiber with the polymer matrix [20, 21, 22]. The results of this study show that for these composites, the mechanical properties were relatively low. Although the use of WH fiber as composite reinforcement still requires further studies, it has several advantages which include, increased acoustic, damping ability and good thermal resistance [23].

Previous researchers on the use of water hyacinth fiber as reinforcement composite still uses WH in the form of stem, stem chopped, sawdust, and powder [22, 24, 25, 26]. No research has been found that uses water hyacinth in the form of fiber or woven fibers. The use of natural fiber as a composite reinforcement, the shape of the reinforcement affects the mechanical properties of composite. This research uses water hyacinth in the form of fibers, either unidirectional or woven fibers. WH fiber can be obtained by extracting WH stem. The WH plant extraction process affected the mechanical properties of the fiber composite. This process aims to separate plant fibers from the wax, pectin, hemicellulose and lignin layers. Also, there were several methods used to extract plant fibers from the parent plant, namely: immersion, chemical methods or mechanical methods [27]. Therefore, this study aims to investigate the mechanical and physical properties of composites reinforced with unidirectional and woven WH fibers.

## 2 Materials and methods

### 2.1 Materials

**Figure 1:**
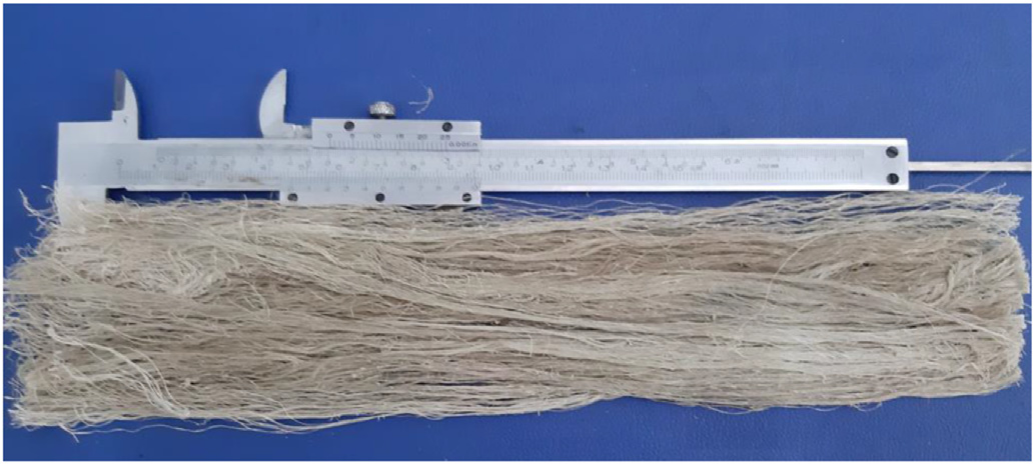
Water hyacinth fibers.

**Figure 2:**
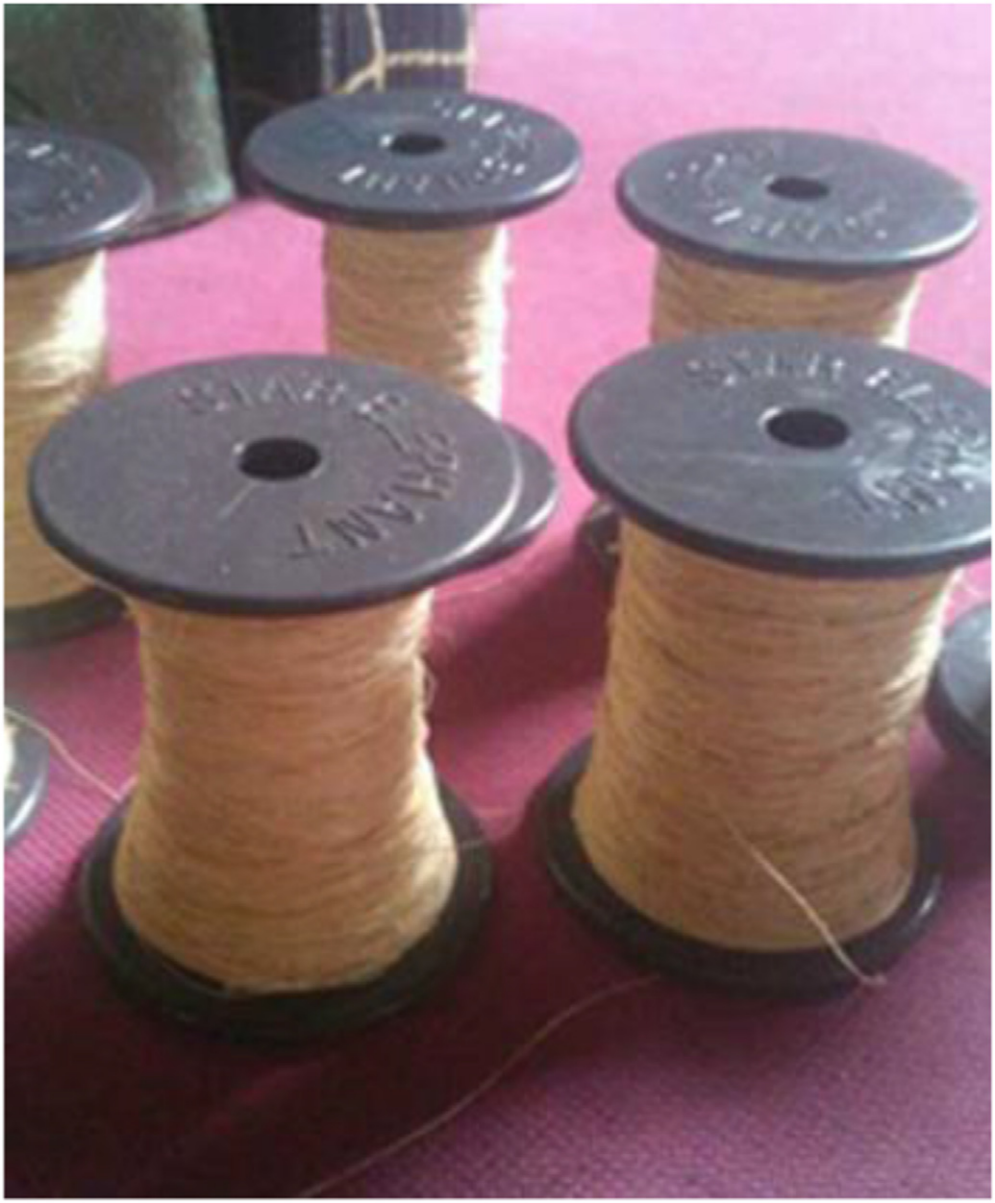
Water hyacinth yarn.

**Figure 3:**
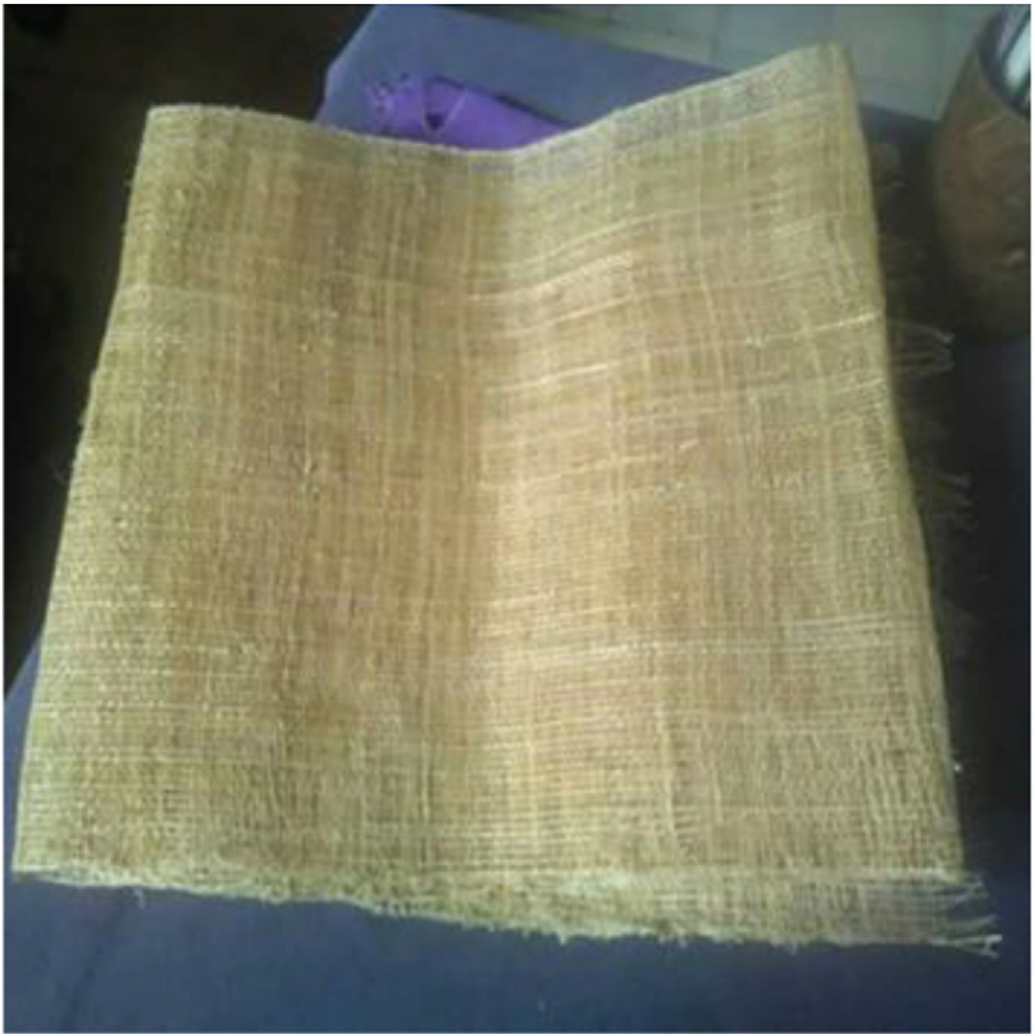
Water hyacinth woven yarn.

The water hyacinth plants used, were obtained from swamps in Tanggul Village, Mijen District, Demak Regency, Central Java, Indonesia with 50–70 cm length of stems. These plants were mechanically extracted to yield the WF fibers (Figure 1). The fiber extraction was carried out by brushing the WH stems using an iron brush. The fiber is then dried in the sun. After that, 10 strands of the dry WH fibers were twisted to produce yarn (Figure 2). The water hyacinth yarns were prepared by yarn craftsmen using a traditional yarn spinner. The WH yarns were then woven using a loom. The weaving process was carried out by traditional cloth craftsmen. This process produced WH mat with dimensions of 30 × 40 cm with direction of yarn 0°/90° (Figure 3). The natural composite was manufactured using epoxy Bakelite® EPR 174 and resin hardener V-140 as a matrix.

### 2.2 Preparation of composites

The hand lay-up method was used to fabricate the unidirectional WH fiber composite and woven WH composites. This was conducted by applying an epoxy-resin matrix to the WH fibers in the mold specimens with dimension of 30 × 40 cm using a paintbrush and roller. The composite tensile and impact test specimens were manufactured in variations of the WH fiber reinforcement of 0%, 15 %, 25 % and 35 % wt. The coding of these specimens is shown in Table 1.

**Table 1:** Specimens testing code.

Code	Meaning
E(0)	Epoxy – resin, 0 % WH fibers
E(15)	Epoxy – resin, 15 % WH fibers
E(25)	Epoxy – resin, 25 % WH fibers
E(35)	Epoxy – resin, 35 % WH fibers

Afterward, the composites were compressed and vacuumed to remove voids and obtain a smoothen surface. After removing the produced composite specimen from the mold, specimens of appropriate dimensions were made in accordance with ASTM D3039 requirements. Water jet cutting technique is used for cutting the test specimen as per the required shape. The test specimen having dimension of length 250 mm, width of 25 mm and thickness of 3 mm were prepared for tensile testing.

### 2.3 Characterization of composites

The actual density of composites was measured using a densimeter based on Archimedes law according to ASTM D792. According to ASTM D2734 the porosity can be obtained by the relative difference between theoretical density of composite and measured density of composite (ρm). The density of composite (ρm) can be measured through water buoyancy by Archimedes Principle (ASTM D792). This leads to the following equation for calculation of the porosity (eq. 1).

(1) Porosity = 100 – ρm{(Wr/ρr) + (Wf/ρf)}

where W and ρ represent the weight percentage and the density, and r and f stand for resin and fiber, respectively.

**Figure 4:**
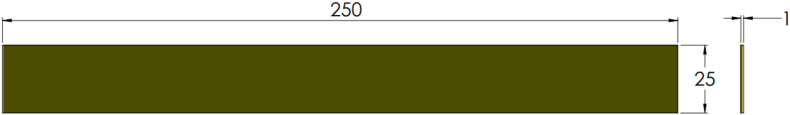
Dimension of tensile test specimen.

**Figure 5:**
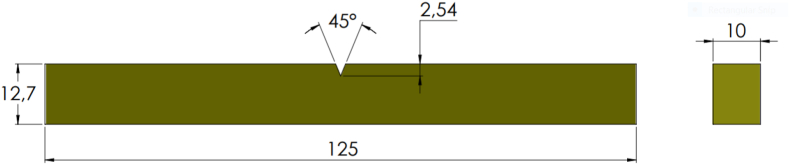
Dimension of impact test specimen of composite.

The tensile test of composites was carried out according to the ASTM D3039 standard (Figure 4). Furthermore, the impact tests were carried out through the Charpy methods according to the ASTM D6110 standard. Figure 5 shows the dimensions of the impact test specimens. Six identical test specimens were prepared for tensile and impact test to ensure the uniformity of the test. Scanning Electron Microscopy (SEM) was used to investigate the tensile test fracture surface of the composite.

## 3 Results and discussions

**Figure 6:**
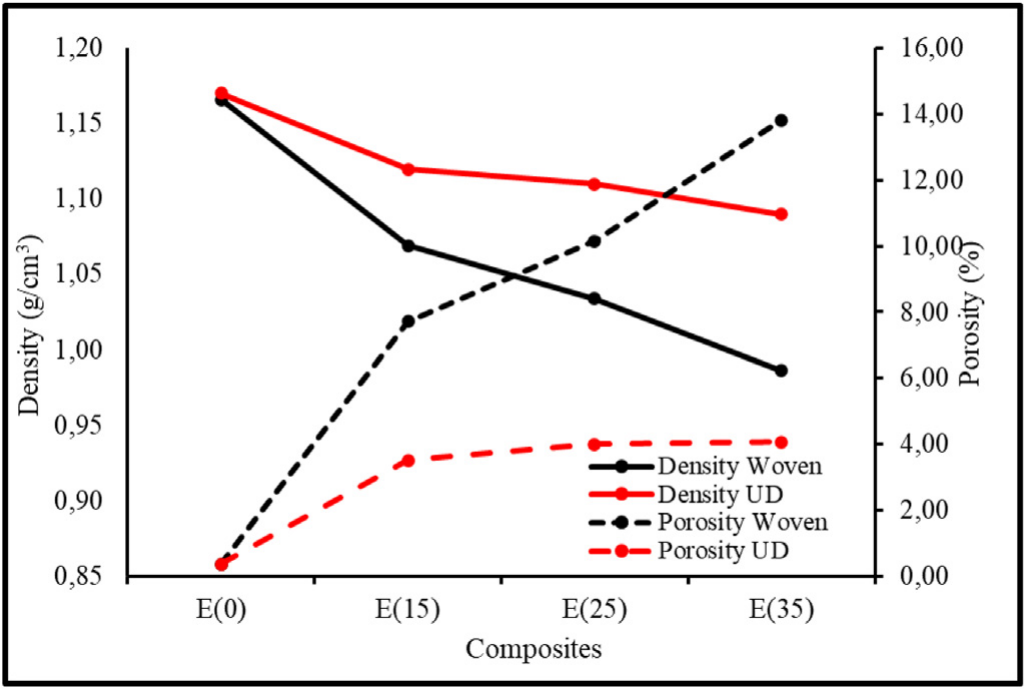
Density and Porosity of WH fibers Composites.

The density and porosity test results in Figure 6, shows that both the unidirectional and woven composites increased in mass fraction, while 0, 15, 25 and 35 % wt. increase in the porosity of the composites was observed. Furthermore, Figure 6 also shows that for the woven fiber composite, there was an increase in porosity almost linearly from 0.35% to 13.82% with the addition of 35% fiber. The unidirectional WH fiber composite, increased from 15 % to 35 % wt., afterwards, the porosity did not increase significantly. Composites woven fibers also provided more voids than composites UD fibers. For woven fibers, the voids form inside tows, in resin-rich regions or at tow corners, and between plies in composites. Meanwhile, for UD fibers, the voids form within and between the plies [28, 29]. The difference in the direction of the fibers for the composites woven fiber leads to air entrapment and increased porosity. The main source of air entrapment is the inhomogeneous fiber architecture, which leads to a non-uniform fiber permeability carried out with subsequent local variations in resin velocity [30].

**Figure 7:**
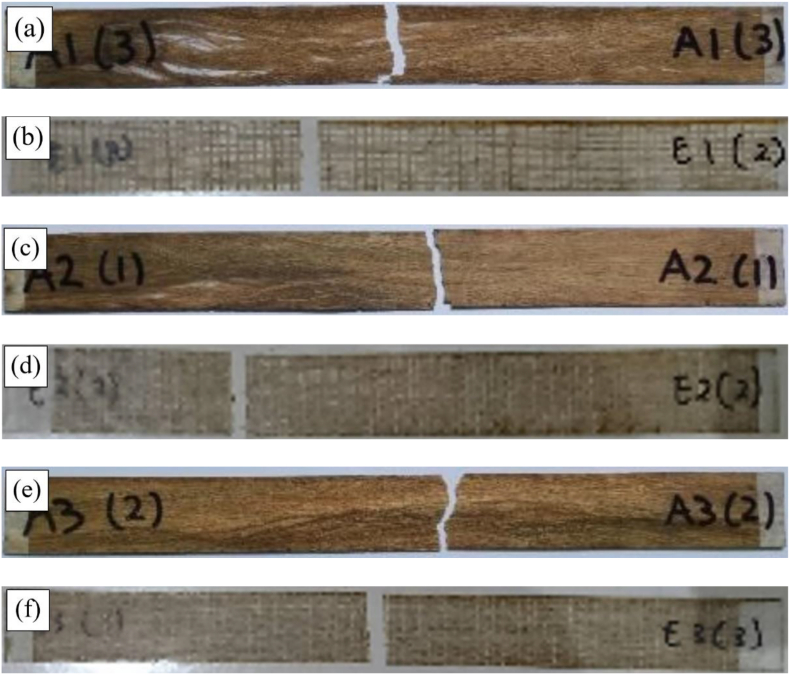
Fracture specimens (a) 15 % unidirectional WH fiber composite. (b) 15 % WH woven fiber composite (c) 25 % unidirectional WH fiber composite (d) 25 % WH woven fiber composite (e) 35 % unidirectional WH fiber composite (f) 35 % WH woven fiber composite.

**Figure 8:**
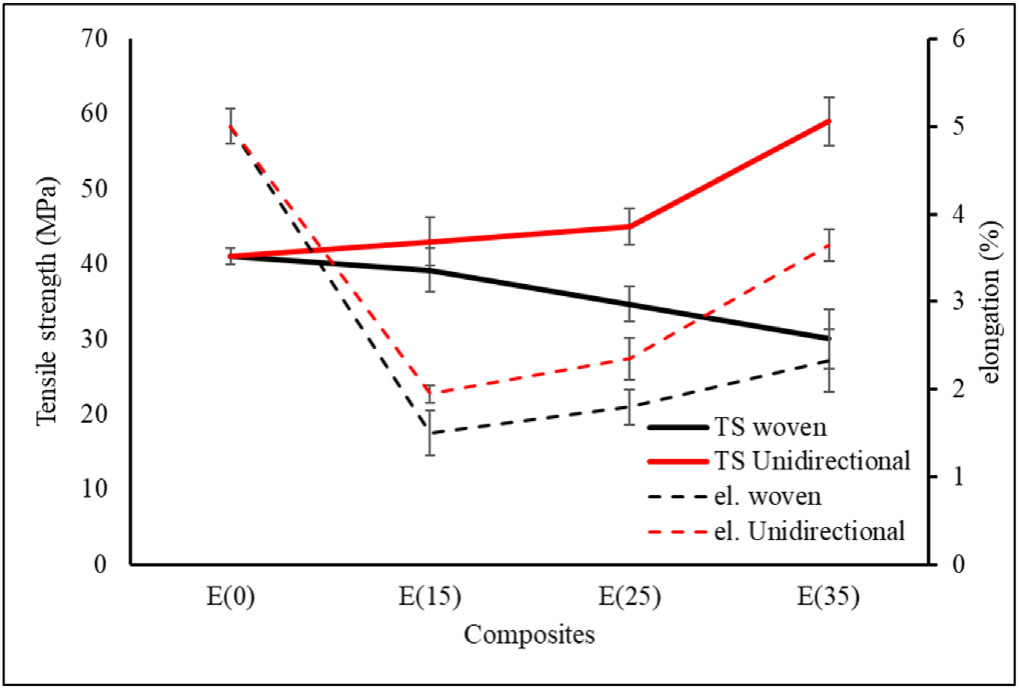
Tensile strength and elongation WH Fibers Reinforced Epoxy-Resin.

**Figure 9:**
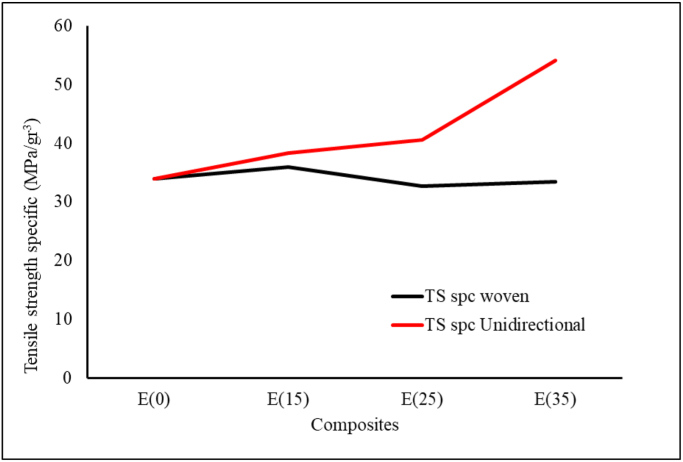
The specific tensile strength of WH Fiber Reinforced with Epoxy-Resin.

Figure 7 shows the specimen after it was tensile tested, with fracture in the gauge length area. Figure 8 shows the tensile test results of the woven and unidirectional WH fiber composites reinforced with epoxy resin. The tensile strength of the woven WH fiber composite was in inverse proportion to the percentage of WH fibers in the composite. An increased fiber mass fraction from 0 to 15%, led to an insignificant decrease in the tensile strength of the composite. Furthermore, with a 35% addition, for every 10% increase in fiber mass fraction, the tensile strength of the composite decreases by about 11–13%. Composite elongation also decreased with the increase in % wt. of WH woven fibers, which also showed that its tensile strength was in direct proportion to the percentage weight of the WH fiber. This tensile strength increased by approximately 10% as the % wt. of WH fibers increased from 15% to 25%. For addition of 35% wt. of WH fiber, the tensile strength of composites increased by about 37%. Compared to the tensile strength of epoxy resin, the composite tensile strength of 35 % wt. for unidirectional WH fiber increased from 41 MPa to 60 MPa or by about 46 %. Furthermore, the lowest composite tensile strength for WH fibers in this study was 30 MPa for woven WH fibers composites containing 35% wt. This result was higher than that of Saputra et al., which discovered that the highest tensile strength was 28.36 MPa [31]. Figure 9 shows the specific tensile strength of composites, which shows that there were no significant differences in the tensile strengths of WH woven fibers composites. Furthermore, reinforcement by WH fibers compensates for the decreasing tensile strength of the composite from porosity. The UD WH fiber composites of 35 % wt., increases in specific tensile strength by about 60 %, which means that, it is in direct proportion with the specific tensile strength of composites.

The single fiber of water hyacinth has a tensile strength of around 105–313 MPa [32]. The results of the tensile test show that woven water hyacinth did not provide an effect of increasing strength on uniaxial tensile loads. Furthermore, the increased mass percentage of the WH reinforcing fibers led to increased composite porosity. A larger volume of woven reinforcing fibers leads to an increase in voids [30, 33], due to the pores being trapped causing a void between the matrix and fibers [34]. The increasing porosity also caused by the incomplete infiltration of resin in the hand lay-up process. Voids of composite produce stress concentration on the matrix. For the longitudinal fibers, voids lead to the potential change in stress transfer and redistribution and the cracking of the transverse plies [28]. The voids between fiber and matrix lead to a fiber pull-out fracture, as shown in Figures 11(a), 11(b), and 11(c).

Composites had an increased tensile strength due to the unidirectional properties of the WH fiber. The unidirectional fiber has a higher percentage of fiber in the direction of tensile load than woven fiber. Based rule of mixture theory, higher % wt. of fiber on axis direction of load yields higher values of tensile strength than the combination of axial and transversal fiber direction in woven fiber. Additionally, an increase in the percentage weight of these fiber composites also improves tensile strength. This is coherent with the density and porosity test results in Figure 6, which shows that the composites with directional woven fiber exhibited a significant increase in porosity as the fiber percentage weight is increased. The porosity of UD WH fiber composites remained constant as the weight percentage of WH fibers increased. Hence, composites with directional woven fiber increased in % wt. and porosity significantly.

**Figure 10:**
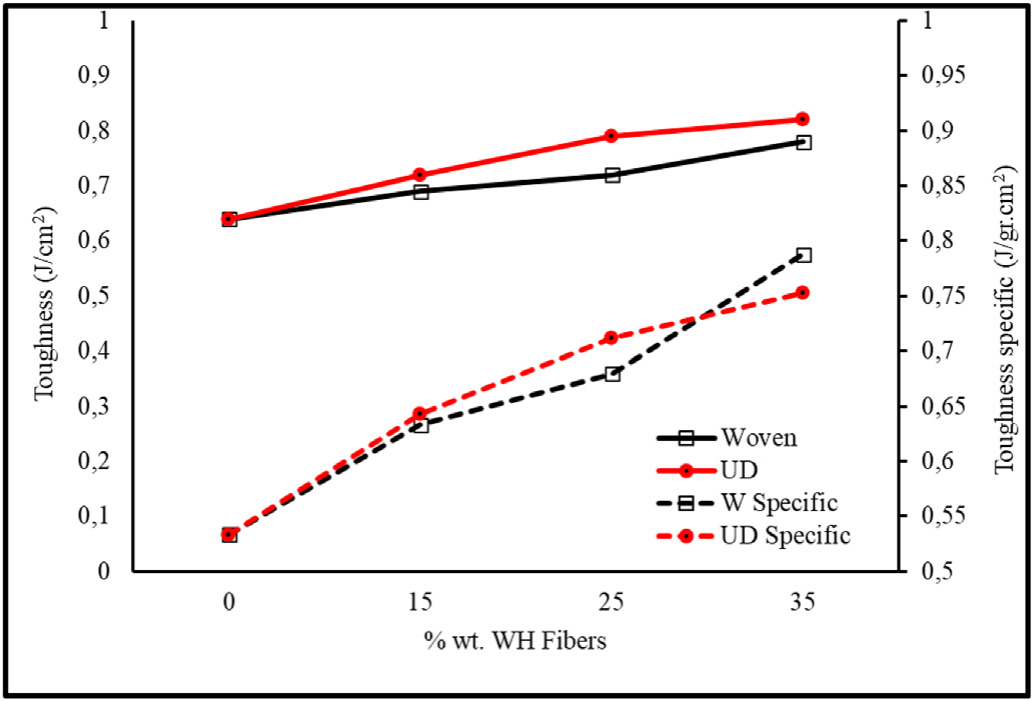
Impact Strength of Composites WH Fibers reinforced Epoxy-Resin.

Figure 10 shows the impact strength of WH fiber reinforced with epoxy-resin. This composite strength was in direct proportion to WH fiber rise of % wt. Increasing of % wt. of UD fiber from 0 % to 35 % wt., increase the impact strength of composite about 28 %. The impact strength of woven fiber composite increases about 22 % when the % wt. of fiber increase from 0 % to 35 % wt. The highest impact strength is about 0.82 kJ/cm^2^ at the UD fiber composite with 35 % wt. of fiber. Figure 10 also shows specifics impact strength of composite. Graph in Figure 10 shown that specific impact strength of composite with 35 % wt. woven WH fiber higher than specific impact strength of 35 % wt. of UD composite. The porosity on woven composite produced lighter composite than UD composite. Woven WH fibers increase the impact strength of the composite and produce a tougher composite. Woven WH fibers have good ability to absorb large share kinetic energy so fibers role should be crack stopper [35]. The UD composite fibers break at the impact axis and the failure of the woven specimen is caused by shear stress [36]. The increase in composite impact strength is due to the interface strength between the woven WH fiber and epoxy - resin matrix contributes to the transfer load from the matrix fiber. This characteristic allows the WH fiber composites to absorb more energy. The fiber flexibility that slides out of the matrix did not break but increased the energy needed to rupture the specimen [37].

**Figure 11:**
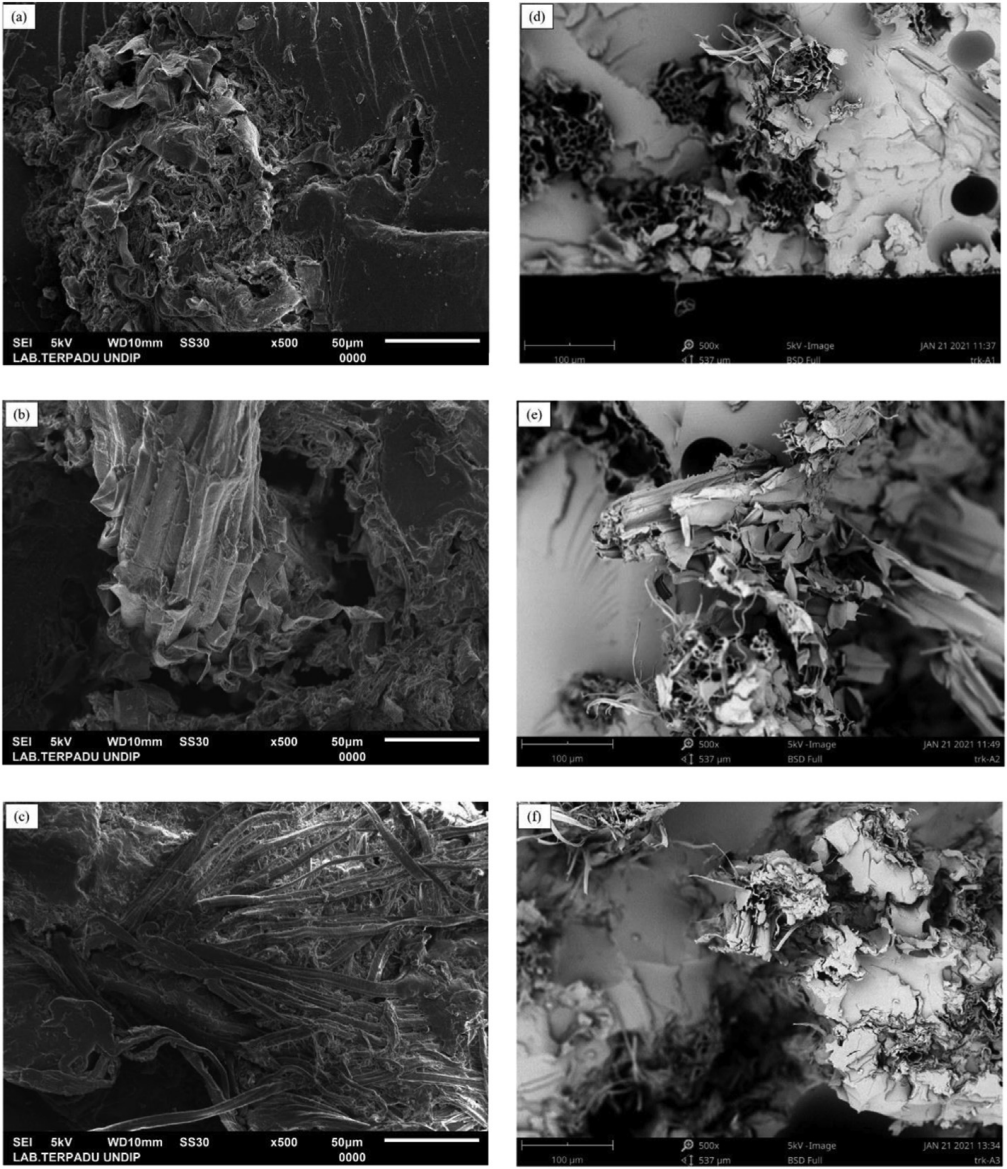
SEM Micrograph Surface Fracture of Composites (a) 15 % WH woven fiber composite (b) 25 % WH woven fiber composite (c) 35 % WH woven fiber composite (d) 15 % unidirectional WH fiber composite (e) 25 % unidirectional WH fiber composite (f) 35 % unidirectional WH fiber composite.

Furthermore, the SEM photomicrographs of the fracture surface were used to analyze the WH fiber and matrix adhesion in the composites. The cross-section of these tensile-tested specimens was selected for analysis. Figure 11a – 11. f shows the fracture surface SEM images of the WH fiber-reinforced epoxy resin composite. Meanwhile, Figure 11a, 11b and 11. c shows the samples with 15, 25, 35 % of WH woven fiber. Figure 11d, 11e and 11. f shows the composite with 15, 25, 35 % of UD WH fiber. From the fractographic examinations by SEM, the fracture behavior of composite was brittle. Some pores, which originated from the fiber pull-out phenomena were found in all cases, while fracture phenomena were also observed in the UD WH fiber. SEM images showed that the WH fiber and epoxy resin were mechanically bound, however, there was no chemical bonding between the WH fiber and epoxy resin. Furthermore, similar natural fiber and polymer adhesion were experimented with and discussed within natural fiber-reinforced plastic composites [38, 39]. For this reason, the mechanical properties of UD WH fiber epoxy resin composites had a 60% increase.

## 4 Conclusion

Previous researchers that used water hyacinth fiber as a composite reinforcement in the form of stem, stem chopped, sawdust, and powder produced mechanical properties under the tensile strength of the matrix material. The use of UD WH fiber as reinforcement results in an increasing of strength of composite. The lowest tensile strength produced in this research is also higher than the highest tensile strength produced by previous researchers. Increasing the % wt. of the WH woven fibers decreased the tensile strength of the epoxy resin composites. Furthermore, the % wt. of WH woven fibers was in direct proportion to the number of pores or voids between the fibers and matrix which led to a delamination mode fracture. The rise of % wt. of fibers increases the tensile and impact strength of unidirectional WH fibers epoxy resin composite. The utilization of WH fibers obtained an increased effective reinforcement. The impact strength of composites was in direct proportion to the rise of % wt. of WH woven fibers. The mechanical properties of unidirectional WH fiber higher than mechanical properties of woven WH fiber composite.

## Declarations

### Author contribution statement

S. Sulardjaka: Conceived and designed the experiments; Performed the experiments; Analyzed and interpreted the data; Contributed reagents, materials, analysis tools or data; Wrote the paper.

N. Iskandar: Performed the experiments; Analyzed and interpreted the data.

Sri Nugroho: Contributed reagents, materials, analysis tools or data; Wrote the paper.

A. Alamsyah, M. Y. Prasetya: Analyzed and interpreted the data.

### Funding statement

This work was supported by the 10.13039/501100005980Kementerian Pendidikan dan Kebudayaan (contract number: 225-108/UN7.6.1/PP/2021).

### Data availability statement

Data will be made available on request.

### Declaration of interests statement

The authors declare no conflict of interest.

### Additional information

No additional information is available for this paper.
